# Human Summating Potential Using Continuous Loop Averaging Deconvolution: Response Amplitudes Vary with Tone Burst Repetition Rate and Duration

**DOI:** 10.3389/fnins.2017.00429

**Published:** 2017-07-27

**Authors:** Alana E. Kennedy, Wafaa A. Kaf, John A. Ferraro, Rafael E. Delgado, Jeffery T. Lichtenhan

**Affiliations:** ^1^Department of Communication Sciences and Disorders, Missouri State University Springfield, MO, United States; ^2^Department of Hearing and Speech, University of Kansas Medical Center Kansas City, KS, United States; ^3^Department of Biomedical Engineering, University of Miami Coral Gables, FL, United States; ^4^Department of Otolaryngology, Washington University School of Medicine St. Louis, MO, United States

**Keywords:** cochlea, auditory nerve, phase locking, tone burst, high stimulus rate, continuous loop averaging deconvolution

## Abstract

Electrocochleography (ECochG) to high repetition rate tone bursts may have advantages over ECochG to clicks with standard slow rates. Tone burst stimuli presented at a high repetition rate may enhance summating potential (SP) measurements by reducing neural contributions resulting from neural adaptation to high stimulus repetition rates. To allow for the analysis of the complex ECochG responses to high rates, we deconvolved responses using the Continuous Loop Averaging Deconvolution (CLAD) technique. We examined the effect of high stimulus repetition rate and stimulus duration on SP amplitude measurements made with extratympanic ECochG to tone bursts in 20 adult females with normal hearing. We used 500 and 2,000 Hz tone bursts of various stimulus durations (12, 6, 3 ms) and repetition rates (five rates ranging from 7.1 to 234.38/s). A within-subject repeated measures (rate x duration) analysis of variance was conducted. We found that, for both 500 and 2,000 Hz stimuli, the mean deconvolved SP amplitudes were larger at faster repetition rates (58.59 and 97.66/s) compared to slower repetition rates (7.1 and 19.53/s), and larger at shorter stimulus duration compared longer stimulus duration. Our concluding hypothesis is that large SP amplitude to short duration stimuli may originate primarily from neural excitation, and large SP amplitudes to long duration, fast repetition rate stimuli may originate from hair cell responses. While the hair cell or neural origins of the SP to various stimulus parameters remains to be validated, our results nevertheless provide normative data as a step toward applying the CLAD technique to understanding diseased ears.

## Introduction

Electrocochleography (ECochG) is a technique that can be used to objectively assess physiologic properties of the auditory periphery. The application of ECochG to both clinical and research purposes is extensive and its use as a diagnostic tool for Ménière's disease has long been considered. While specific criteria have been examined, such as the use of the summating potential (SP)/compound action potential (AP) amplitude ratio, the relatively low sensitivity of this measure alone has limited its diagnostic value for Ménière's disease (Ferraro and Tibbils, 1999; Ferraro and Durrant, 2006; Al-momani et al., 2009). The lack of sensitivity of the SP/AP ratio measure obtained from click stimuli, and the unknown origins of the disease, has led to the continued refinement of ECochG uses to advance the differential diagnosis of Ménière's disease.

One such method has been the use of tone burst stimuli to assess the SP across frequencies. As Ménière's disease typically presents with fluctuating hearing loss, initially affecting the low frequencies, physiologic measurements from throughout the length of the cochlear spiral may help provide new insight into the disease. While the origins of various components of the SP and AP components are still being sought after and understood, both have been shown to vary greatly with stimulus parameters. While the SP and AP can interleave in a given measurement, the amplitude of the SP appears to sustain for the duration of the response and makes it an attractive attribute to study.

Gibson (1993) was one of the first to develop criteria for the use of tone burst ECochG measurements to assess of Ménière's disease with the SP amplitude. Gibson (1993) determined that the most effective frequencies when evaluating the disorder were 500 and 1,000 Hz, while 4,000 Hz was the least effective. Gibson (2009) repeated the study with matched hearing loss controls (ears without Ménière's disease, but with sensorineural hearing loss) and found that 500, 1,000, and 2,000 Hz were most sensitive, while significant overlap in responses between groups occurred at 4,000 and 8,000 Hz. (Gibson, 1993, 2009) also compared the results to click stimuli SP/AP amplitude ratio measurements, and determined the use of tone burst SP amplitude was a sensitive measure to Ménière's disease. Others have found increased sensitivity with SP amplitudes obtained from 1,000 Hz tone burst stimuli when compared to click evoked SP/AP amplitude ratios (Conlon and Gibson, 2000; Iseli and Gibson, 2010). These findings support the use of frequency specific stimuli in ECochG measures when examining the effects of Ménière's disease.

At present time, the majority of tone burst ECochG studies designed to examine the SP have used relatively long stimulus durations (≥12 ms). While this approach allows for clearer observation of the SP after the AP amplitude has adapted, it limits the stimulus repetition rate at which tone burst stimuli can be presented without overlaying of the signal. Wuyts et al. (2001) examined the effect of 1,000 Hz repetition rate on the SP amplitude in subjects with and without Ménière's disease. Stimulus repetition rate was varied between 8.4 and 37.4 tone bursts/second and the investigators found that that SP amplitude increased with increased rate, regardless of the presence or absence of the disease, with larger SP amplitude found in those with the disease. While Wuyts et al. (2001) studied the effect varying stimulus repetition rate using transtympanic ECochG, there is limited research focused on the use of extratympanic ECochG measurements to tone burst with various repetition rates, particularly above 37 tone bursts/second.

The use of high stimulus repetition rates face limitations as rate increases to the point where the responses overlay, obscuring one another. As measurements to high rates are significantly degraded and difficult to interpret using the standard measure analysis technique, ECochG to very high repetition rates requires a special technique to help analyze the complex, overlain responses (Delgado and Ozdamar, 2004). This complex waveform occurs as the response from one eliciting stimulus has not ended before the presentation of the next. Recently, a new technique, continuous loop averaging deconvolution (CLAD), has been designed to employ algorithmic formulas to deconvolve or “unmix” waveforms collected at very high rates. The application of CLAD to ECochG measurements obtained with high stimulus repetition rates has been utilized successfully. Kaf et al. (2017) quantified normative ECochG and ABR measures to click at rates up to 507 clicks/s using this novel technique. The CLAD technique has also shown promise in the assessment of Ménière's disease through the use of high, 780 clicks/s, repetition rates (Bohorquez et al., 2009).

The present study was designed to investigate the effects of high rate and stimulus duration on SP amplitude of 500 and 2,000 Hz tone burst ECochG in adults with normal hearing. This research is the first step in understanding the physiological effect of high rate on tone burst ECochG in subjects without a history of inner ear pathology, and in establishing normative SP amplitude data upon which further research can build. The goals of this study are to (1) establish normative SP amplitude data for high repetition rate 500 and 2,000 Hz tone bursts, and (2) quantify the effect of stimulus duration on measurements to various stimulus repetition rates.

## Methods

### Participants

This study was approved by the Missouri State University Institutional Review Board and written informed consent was obtained from each participant. Twenty-one female adults between the ages of 20–35 years with normal hearing sensitivity were recruited for participation in this study. However, due to poor replicability of tone burst waveforms from one participant, only the data of 20 participants was analyzed in this study. Criteria for participation in the study included: (1) otoscopic evaluation revealing ear canals clear of cerumen and debris, (2) normal hearing sensitivity determined by pure tone air conduction audiometry, with thresholds ≤25 dB HL from 250 to 8,000 Hz (Goodman, 1965); (3) normal middle ear status as confirmed by 226 Hz tympanometry and the presence of normal static compensated admittance, tympanometric pressure, and ear canal volume (American Speech-Language-Hearing Association, 1988); and (4) a recordable SP and AP with standard click ECochG measurements. Female participants were recruited for participation in this study. Although gender differences were not assessed during this study, previous research has not demonstrated significant differences in ECochG responses between male and female subjects (Wilson and Bowker, 2002).

### Equipment

All participants were tested in the sound booth at the Missouri State University auditory research laboratory. The Intelligent Hearing Systems SmartAudiometer was used to assess hearing thresholds from 250 to 8,000 Hz using pure tone stimuli presented via ER-3A insert earphones under sound booth conditions. The Intelligent Hearing System Smart–Evoked Potential equipment was used for the extratympanic ECochG recordings; with ER-3A insert earphones used to deliver the stimuli. The equipment was calibrated according to manufacturer specifications, using a precision sound level meter (Quest, Model 155), microphone (Bruel and Kjaer, Model 4144), and a 2-cc (HA-2) coupler (Bruel and Kjaer, Model DB-0138) and followed the IEC standard for peSPL (0 dBnHL = 32 dB peSPL ±3 dB). A homemade tympanic electrode (Ferraro and Durrant, 2006) was used as the inverting electrode placed on the tympanic membrane. The materials used to construct the electrodes included bare silver wire (0.008 inch diameter), silicon tubing (0.0077 inch outer diameter; 0.058 inch inner diameter), cotton balls, electrode conducting gel, and a needle syringe. A microalligator clip was used to connect the wire end of the tympanic membrane electrode to the pre-amplifier.

### Stimulus and recording parameters

A one channel montage was used for ECochG recording from the test (right) ear of each participant. The inverting tympanic membrane electrode was placed on the tympanic membrane of the right ear, the non-inverting electrode was placed on the ipsilateral (right) mastoid, and the ground electrode was placed on the contralateral (left) mastoid. Ferraro et al. (1994a) suggest the use of an ipsilateral montage in order to reduce the contribution of later waves associated with ABR in the response. Electrode impedance was kept ≤7 Ω at each electrode site.

Prior to the collection of tone burst ECochG at high rate, standard, slow rate click ECochG was performed for the right ear. This step allowed for a clear observation of the SP and AP components in the waveform to ensure these potentials were present under standard ECochG parameters prior to the implementation of the experimental test protocol. Hundred microsecond broad-band click stimuli were presented at 75 dB nHL, with alternating polarity at a rate of 7.1/s. The recording epoch was set for 5 ms. A band-pass filter setting of 10–3,000 Hz and a gain setting of 100,000 were utilized. Two traces were collected, each recorded for 1,000 sweeps.

For the present study, the rate values examined included 7.1, 19.53, 58.59, 97.66, and 234.38/s. All rates, with the exception of 7.1/s, are CLAD rate sequences that were developed and evaluated by the Intelligent Hearing Systems for their ability to deconvolve the recorded response using the CLAD algorithm. These four CLAD stimulus rates were chosen based on the stimulus durations of the tone burst stimuli in order to ensure no overlap occurred in the eliciting signal. As stimulus rate is limited by the stimulus duration, higher rates could not be used without the potential of overlap in the stimulus signal which would be detrimental to the recordings. Loopback recordings of the 500 and 2,000 Hz stimuli were performed at each rate to ensure no overlap occurred within the stimulus.

Each trace was repeated to ensure replicability, with the 2,000 sweeps per trace. The recording epoch was set at 12 ms. As with standard ECochG, recordings were made using an alternating polarity signal a gain of 100,000, and were presented at an intensity level of 75 dB nHL (107 dB SPL). The band-pass filter was set to 3–3,000 Hz; a high pass filter of 3 Hz was used because the SP, as a direct current potential, is particularly sensitive to high pass filter settings. The use of a high pass filter of 3 Hz, is thought to minimize the distortion present in the SP signal (Ferraro et al., 1983).

To examine the effect of rate on SP amplitude response as a function of stimulus duration, recordings were conducted with stimulus durations of 12, 6, and 3 ms for each rate in which no overlap would occur. For example, at 19.53/s all durations (12, 6, and 3 ms) were examined as no overlap occurs at this rate. On the other hand, at the highest rate, 234.38/s, only the 3 ms stimulus duration was examined due to the stimulus overlap that would result from testing using the longer duration stimuli. For both the 500 and 2,000 Hz conditions, 2 ms rise and fall times with an 8 ms plateau was used for the 12 ms duration stimuli and 2 ms rise and fall times with a 2 ms plateau was applied for the 6 ms duration stimuli. For the 3 ms duration stimuli, rise and fall times of 1.5 ms were used, with no plateau.

### Procedures

**Figure 1:**
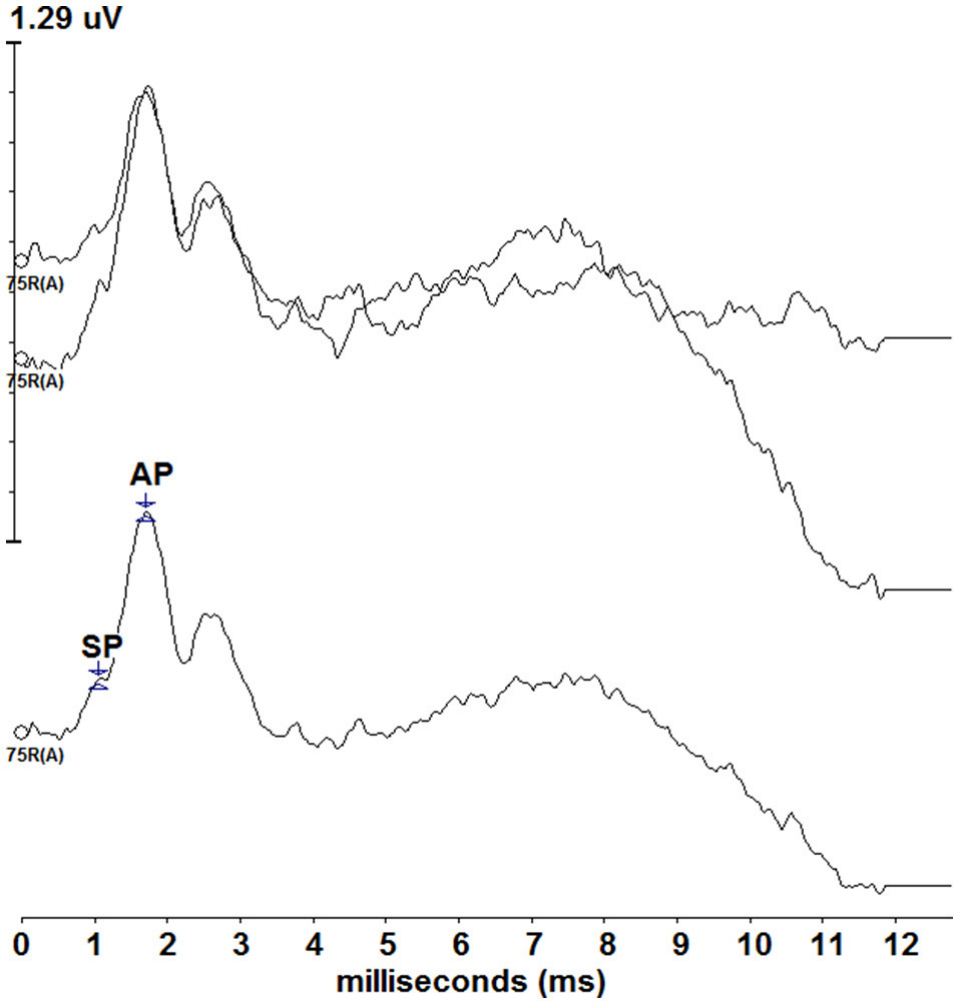
Standard click ECochG responses from one of the participants (P9). Two traces (top) were recorded and averaged (bottom) depicting a typical ECochG response. The SP and AP are labeled on the averaged tracing, though standard ECochG data was not formally assessed during this study.

Standard click ECochG was performed on the right ear for all participants. Though not formally analyzed, standard ECochG was performed on all participants to ensure reliable and replicable click ECochG could be obtained prior to the collection of tone burst ECochG. Figure 1 displays standard click ECochG traces for one of the participants (P9). Two traces were recorded, averaged and assessed to ensure the presence of both the SP and AP waveform components before proceeding with the experimental, tone burst ECochG protocol. In a laboratory environment, participants were comfortably seated in a reclining chair. The participant's skin was scrubbed gently with Nu-Prep gel on the electrode site areas, the participant's right (M2) and left (M1) mastoids. Disposable surface electrodes were then placed and attached to these sites. Next, the tympanic membrane electrode was inserted along ear canal and slowly moved toward the tympanic membrane. The patient was informed that they would feel a slight pressure as the electrode came in contact with the tympanic membrane. The patient was instructed to provide verbal feedback regarding their comfort and the pressure sensation accompanying the contact of electrode with their tympanic membrane. The electrode placement was guided by otoscopy, patient report of tympanic membrane contact, and electrode impedance measure of less than 7kΩ. Following placement of the tympanic membrane electrode, an ER-3A insert earphone was placed in the ear canal to hold the electrode in place and deliver the sound stimuli. The portion of the electrode protruding from the ear canal was taped down to the side of the participant's face and attached to a microalligator clip. Participants were reclined, instructed to relax, and encouraged to take a nap during standard click ECochG and experimental tone burst ECochG testing to 500 and 2,000 Hz.

Following recording of standard click ECochG, tone burst ECochG to 500 and 2,000 Hz were recorded. The order of the tone burst stimuli and the repetition rates were randomized a priori to eliminate any order effect. At each repetition rate, the appropriate stimulus durations were adjusted from long to short duration as applicable. With each duration and rate, two traces of 2,000 sweeps each were recorded. Once all recordings from the right ear at both 500 and 2,000 Hz were completed, the tympanic membrane electrode was removed from the participant's ear and otoscopy was performed to rule out any sign of injury to the ear canal and tympanic membrane as a result of tympanic membrane placement and to assess tympanic membrane contact location.

### Data analysis

Recordings were completed on 21 participants; however, only data from 20 participants were included in the analysis. Data from one of the participants was excluded due to poor replicability of the tone burst ECochG waveforms. In addition, data from one participant for the 500 Hz, 234.38/s condition was excluded from the analysis due to an incomplete recording for that rate. All other recordings were included in the data analysis. Analysis of the recorded waveforms occurred offline. The two recorded, non-deconvolved traces from each condition were averaged (see Kaf et al., 2017; Figure 1 non-deconvovled ECochG to high click rates). Because of the complexity of the non-deconvolved waveforms, the averaged waveforms were then deconvolved using the CLAD algorithm, and the resulting deconvolved traces were labeled to determine the SP amplitude. Uniform labeling was used across all deconvolved waveforms according to the frequency and duration of the recording; rate was not a factor in the labeling of the waveforms. The SP amplitude measurements were made from the midpoint of the stimulus duration, beginning at the onset of the response, to the baseline. SP amplitude measurement from the midpoint of the response is a common practice in the recording of tone burst ECochG and is thought to allow for SP measurement to made without contribution from the AP at the onset of the response and prior to SP decay at the end of the response (Gibson, 1993, 2009; Ferraro et al., 1994a,b; Wuyts et al., 2001). For 12, 6, and 3 ms stimulus durations, the midpoints were 6, 3, and 1.5 ms respectively. Each of these midpoint measures were made from the onset, the beginning of the response, in order to maintain a uniform SP midpoint latency from which the SP amplitude was measured. The onset of the response was chosen as the point at which a positive shift from baseline was noted and was defined as a latency of 1.5 ms for the 2,000 Hz condition, and at a latency of 2.5 ms for the 500 Hz condition across the recordings from all participants. These latency differences between frequencies may be associated with cochlear travel time, which is longer at apical, low frequencies than basal, high frequencies (Ferraro et al., 1994a). All baseline measurements were made at a latency of 1 ms to measure SP amplitude from a point prior to the onset of the response.

Repeated measures analysis of variance was conducted to compare the effect of rate, duration, and the combination of the two on SP amplitude for both the 500 and 2,000 Hz conditions. A 3 (rate—7.1, 19.53, 58.59/s) × 3 (duration—12, 6, 3 ms) within-subject design was utilized in order to assess the interaction across the variables. To evaluate the remaining rates 97.66 and 234.38/s rates, separate one-way analysis of variance for each duration was conducted to compare responses as a function of repetition rate for both frequencies examined. This included comparing three rates (7.1, 19.53, 58.59/s) at 12 ms durations, four rates (7.1, 19.53, 58.59, 97.66/s) at 6 ms durations, and five rates (7.1, 19.53, 58.59, 97.66, 234.38/s) for the evaluation of 3 ms durations.

## Results

**Figure 2:**
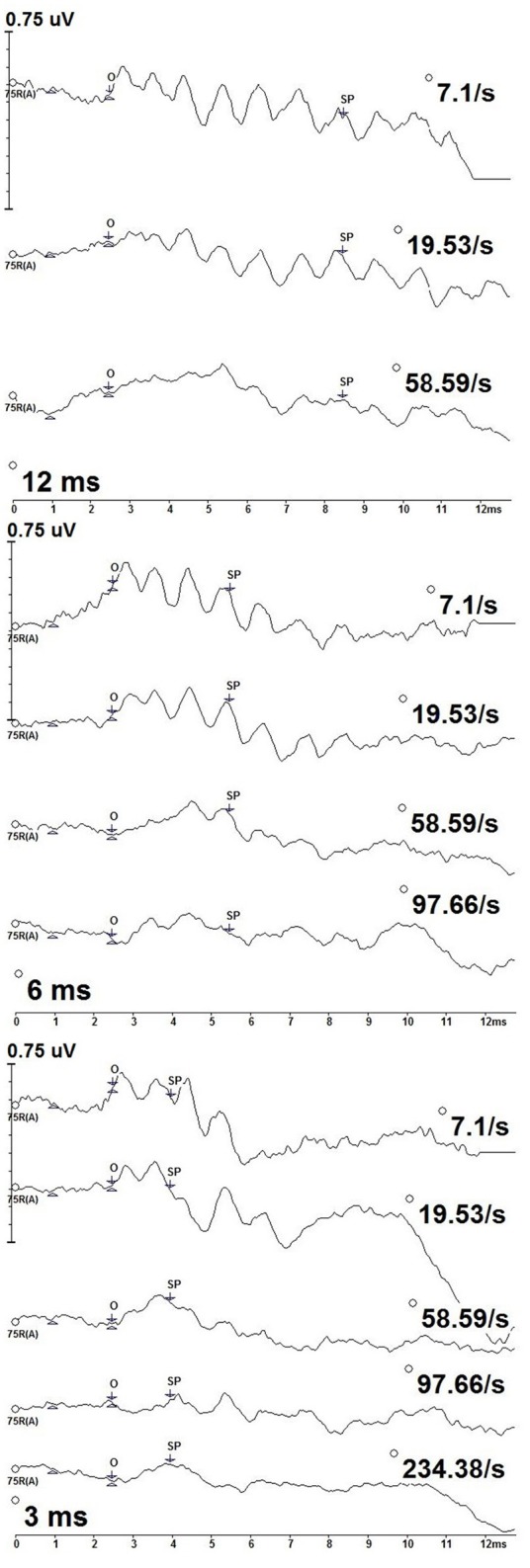
CLAD deconvolved, 500 Hz tone burst ECochG measurements from one participant (P9) across stimulus duration (12 ms—top; 6 ms—middle; 3 ms—bottom). Responses to increasing repetition rate are displayed from top to bottom in each panel. SP amplitude was measured as the baseline at 1 ms to the SP waveform midpoint (SP). The SP waveform midpoint (SP) was measured from onset (O).

Figure 2 depicts the deconvolved tone burst ECochG responses from one of the participants (P9) for the 500 Hz condition for the 12, 6, and 3 ms durations. The SP onset to 500 Hz began at a ~2.5 ms, the location of a positive shift in amplitude from the baseline, and was defined as the starting latency from which the SP midpoint was measured. SP amplitudes were compared across stimulus duration and rate. As the SP is dependent on stimulus duration, the latency of the SP response varied with duration: SP latencies were progressively shorter with decreasing stimulus durations. The SP with the longest latency was to 12 ms stimulus duration, while the shortest was to 3 ms.

SPs differed with stimulus repetition rate duration, particularly to the slowest and highest rates. Most notably, oscillations in the waveform can be observed across the slower rates. This pattern was most evident to slower stimulus repetition rates, 7.1 and 19.53/s, but was less evident to increasing rates and not apparent to the fastest rates. This result is consistent with SP oscillations originating from phase-locked neural excitation that adapts to increasing stimulus rate. Oscillations in the SP waveforms did not cause us to deviate from our methods of quantifying SP amplitudes from various stimulus repetition rates and durations.

**Figure 3:**
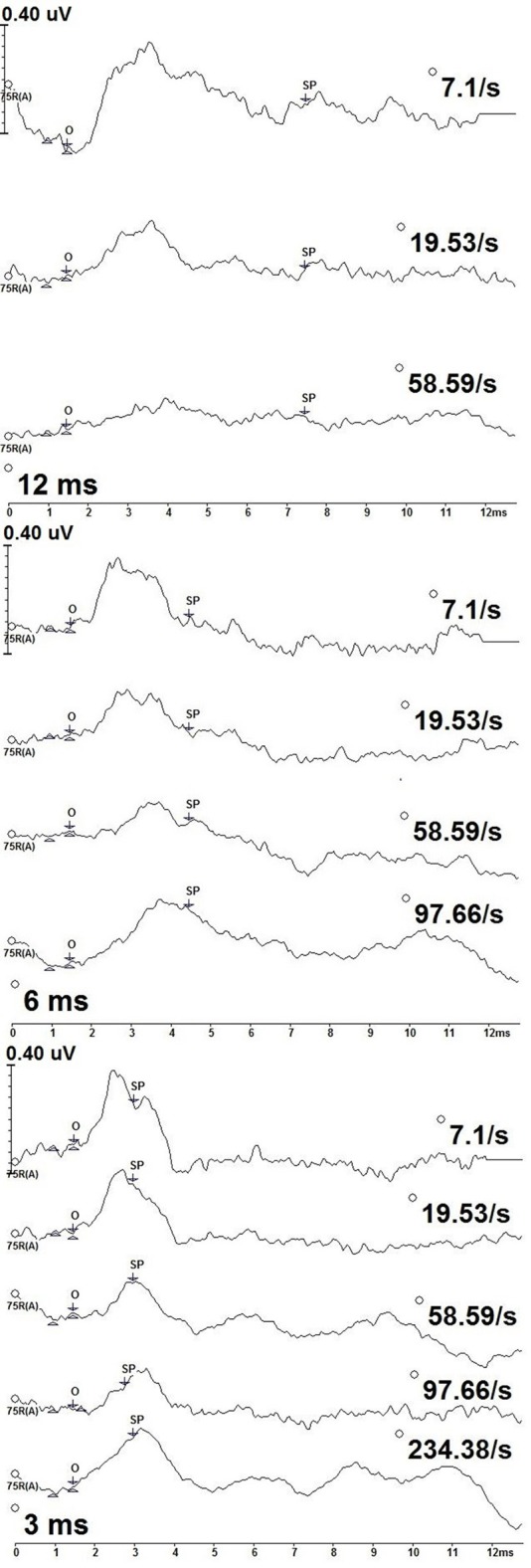
CLAD deconvolved, 2,000 Hz tone burst ECochG measurements from one participant (P9) across stimulus duration (12 ms—top; 6 ms—middle; 3 ms—bottom). Responses to increasing repetition rate are displayed from top to bottom in each panel. SP amplitude was measured as the baseline at 1 ms to the SP waveform midpoint (SP). The SP waveform midpoint (SP) was measured from onset (O).

Figure 3 shows deconvolved measurements from one participant (P9) to the 12, 6, and 3 ms 2,000 Hz stimulus durations. We identified the SP onset (O) as the point where a positive shift from baseline was observed. SP onset was defined as a 1.5 ms latency to 2,000 Hz, an absolute latency kept constant each stimulus repetition rate and duration for all participants. The length of the SP varied with stimulus duration, with the longest SP response associated with the 12 ms stimulus duration and the shortest with the 3 ms duration.

In contrast to the measurements to 500 Hz stimulus, no oscillating patterns were seen in measurements to 2,000 Hz. Rather, the SP to 2,000 Hz was a notable positive amplitude shift from baseline. The SP latency at peak amplitude varied with stimulus repetition rate and duration, with earlier peak SP amplitude latencies being earlier for slower stimulus repetition rates compared to higher rates. Following this peak the SP amplitude was a gradual decrease in amplitude and increasing latency as the measurement approached baseline. While general amplitude trends were observed over the entirety of the waveform, only the amplitude at midpoint of the SP response was formally assessed.

**Figure 4:**
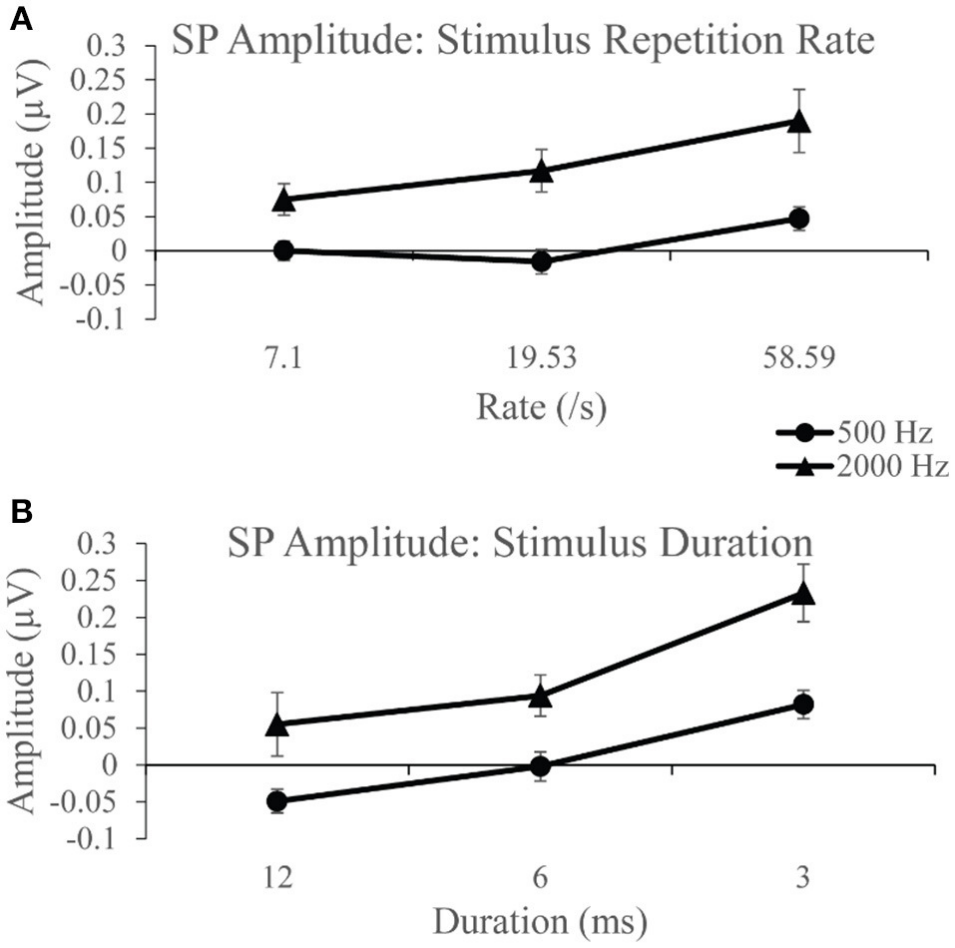
**(A)** SP amplitudes to 500 and 2,000 Hz varied with stimulus repetition rate (7.1, 19.53, and 58.59/s). SP amplitude to both 500 and 200 Hz were larger for 58.59/s compared to rates 7.1 and 19.53/s. **(B)** SP amplitudes to 500 and 2,000 Hz varied significantly with stimulus duration (12, 6, and 3 ms). SP amplitudes to 500 Hz were significantly larger for 3 ms stimulus duration than for 12 ms durations and 6 ms durations. Likewise, SP amplitudes to 2,000 Hz were significantly larger for the 3 ms stimulus duration than for 12 and 6 ms durations.

Group SP amplitudes to 500 Hz varied with stimulus repetition rate (Figure 4A) and duration (Figure 4B). ANVOA results quantified statistically significant differences for main effect of rate, *F*_(2, 38)_ = 6.216, *p* < 0.05, η^2^ = 0.246, and duration, *F*_(2, 38)_ = 16.097, *p* < 0.001, η^2^ = 0.459, and a significant rate and duration interaction, *F*_(4, 76)_ = 3.461, *p* < 0.05, η^2^ = 0.154. The mean difference between SP amplitude as a function of rate was due to significantly larger SP amplitudes (*p* < 0.05) to 58.59/s (mean = 0.047 μV) than to 7.1/s (mean = 0.00 μV) and 19.53/s (mean = −0.016 μV). No significant difference was found between mean SP amplitudes for the two slowest stimulus repetition rates (7.1 and 19.53/s). The SP amplitude was significantly different (*p* < 0.05) between all stimulus durations. Mean SP amplitude increased with decreasing stimulus duration, in that the smallest mean SP amplitude was found for 12 ms duration and the largest for the 3 ms duration. The SP amplitude trends observed as a function of duration and rate independently indicate significant differences between the applied stimulus parameters in the collection of the SP response, with high rate and short stimulus duration leading to the largest SP amplitude measurements.

SP amplitudes to 2,000 Hz varied with stimulus repetition rate (Figure 4A) and duration (Figure 4B). We found statistically significant differences for main rate effect *F*_(2, 38)_ = 6.774, *p* < 0.005, η^2^ = 0.263, and duration effect *F*_(2, 38)_ = 11.379, *p* < 0.001, η^2^ = 0.375, and an interaction between rate and duration, *F*_(4, 76)_ = 6.480, *p* < 0.001, η^2^ = 0.254. The mean difference between SP amplitude as a function of rate is due to significantly larger (*p* < 0.05) SP amplitude at 58.59/s rate (mean = 0.190 μV) than at 7.1/s (mean = 0.075 μV) and 19.53/s (mean = 0.117 μV) rates. No significant difference was found between mean SP amplitudes for 7.1 and 19.53/s. The SP amplitude was significantly larger (*p* < 0.05) to 3 ms stimulus duration compared to the smaller mean amplitudes to 12 and 6 ms. No statistically significant difference was found between the 12 and 6 ms stimulus durations. Again, significant differences across parameters noted, with SP amplitude values significantly larger for short duration and high repetition rates. Further evaluation of SP amplitudes were performed to examine the effect of stimulus repetition rate for each stimulus duration.

**Figure 5:**
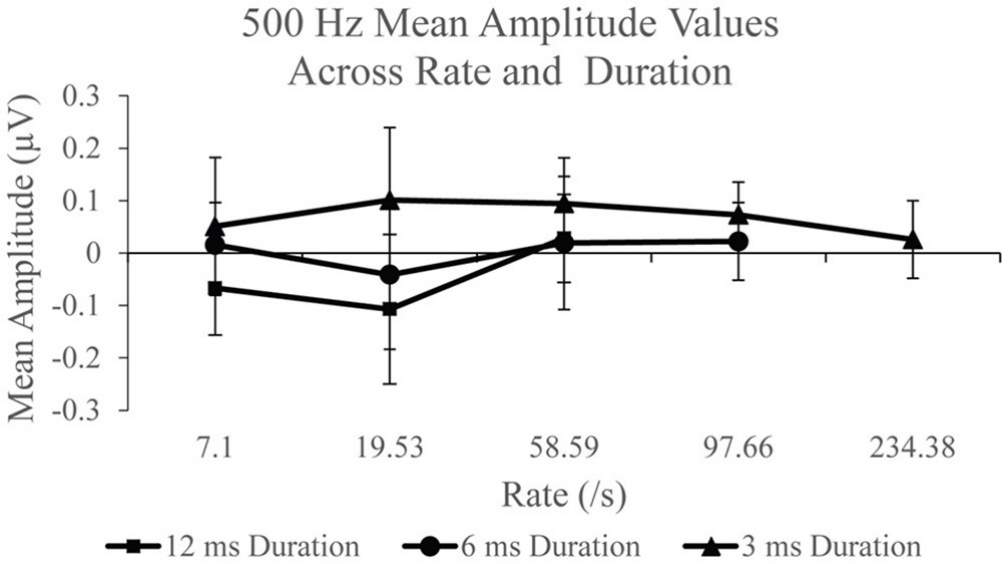
Mean SP amplitude values for the 12 ms duration, 6 ms duration, and 3 ms duration as a function of repetition rate for the 500 Hz condition.

Figure 5 shows mean SP amplitude to each duration of 500 Hz. SP amplitude measurements to each stimulus duration was examined independently across each rate. Statistically significant difference, *F*_(2, 38)_ = 9.74, *p* < 0.005, η^2^ = 0.339 was found for the 12 ms duration across rate. *Post-hoc* pairwise comparison revealed significantly larger (*p* < 0.05) SP amplitude to 58.59/s (mean = 0.028 μV) compared to 7.1/s (mean = −0.067 μV) and 19.53/s (mean = −0.107 μV). No significant difference in SP amplitude was found between rates 7.1 and 19.53/s (*p* > 0.05) to 12 ms stimulus duration. The effect of stimulus repetition rate on SP amplitude was also assessed independently for the 6 ms stimulus duration condition, in order to include 97.66/s, and for the 3 ms duration recordings, in order to include 97.66 and 234.38/s. There was no significant effect (*p* > 0.05) across rate for the 6 ms condition. However, a statistically significant difference, *F*_(4, 76)_ = 2.499, *p* < 0.05, η^2^ = 0.116, was found for the SP amplitude across rate for the 3 ms condition. *Post-hoc* pairwise comparison revealed significant differences (*p* < 0.05) between SP amplitude due to significantly larger SP amplitude to 58.59/s (mean = 0.095 μV) and 97.66/s (mean = 0.07 μV), compared to 234.38/s (mean = 0.026 μV). For the long duration stimuli, a significantly larger SP amplitude is collected with higher stimulus rate; however, the opposite trend is observed with the shorter duration stimuli that produces a smaller mean SP amplitude when the fastest rates are used to elicit the response. To evaluate the SP amplitude data of the responses obtained with use of 2,000 Hz eliciting stimuli, identical analysis procedures were applied.

**Figure 6:**
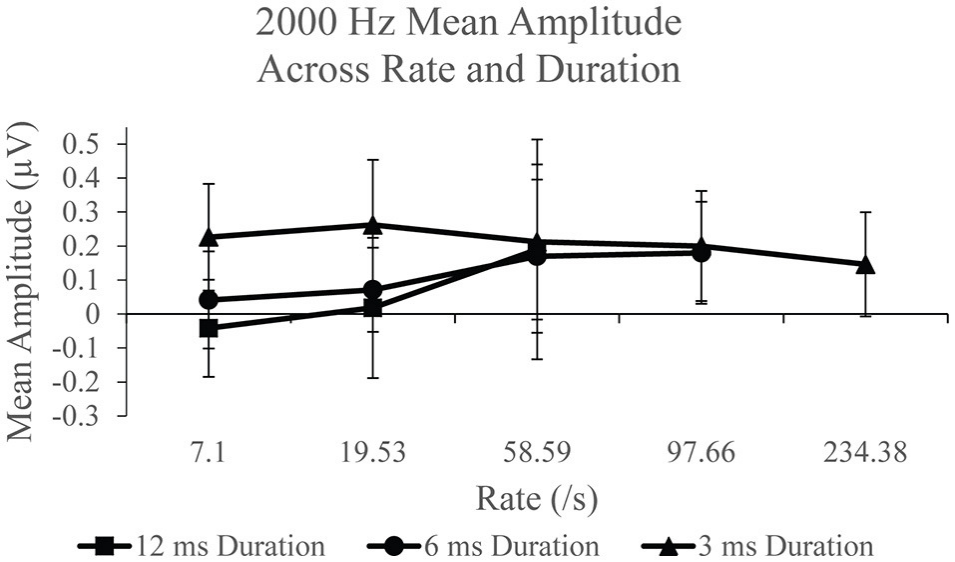
Mean SP amplitude values for the 12 ms duration, 6 ms duration, and 3 ms duration as a function of repetition rate for the 2,000 Hz condition.

SP amplitudes to 2,000 Hz varied with stimulus rate and duration (Figure 6). An independent analysis of the measurements to 12 ms stimulus durations revealed a statistically significant difference across rate *F*_(2, 38)_ = 9.936, *p* < 0.005, η^2^ = 0.343. Significantly larger SP amplitude (*p* < 0.05) was found for 58.59/s (mean = 0.189 μV) compared to 7.1/s (mean = −0.042) and 19.53 (mean = 0.018). No significant difference was found between SP amplitude to 7.1 and 19.53/s (*p* > 0.05) stimulus repetition rates. To examine the remaining rates, 97.66 and 234.38/s, in the analysis, the effect of stimulus rate was assessed independently for the 6 and 3 ms conditions. The 6 ms duration condition revealed a statistically significant difference, *F*_(3, 57)_ = 6.009, *p* < 0.005, η^2^ = 0.240, between SP amplitude across rate. Specifically, a significant difference was found due to larger amplitude at rates 58.56/s (mean = 0.170 μV; *p* < 0.05) and 97.66/s (mean = 0.180 μV; *p* < 0.005), when compared to rate 7.1/s (mean = 0.042 μV). A significantly larger (*p* < 0.005) amplitude was also found for 97.66/s than for 19.53/s (mean = 0.071 μV). No other significant difference (*p* >0.05) was observed between rates for the 6 ms duration. For the 3 ms condition, a statistically significant difference, *F*_(4, 76)_ = 3.384, *p* < 0.05, η^2^ = 0.151, was noted. Specifically, we found a significantly larger (*p* < 0.05) SP amplitude to 19.53/s (mean = 0.262 μV), than for the two highest rates, 97.66/s (mean = 0.201 μV) and 234.38/s (mean = 0.146 μV). No other significant differences (*p* > 0.05) were found between rates for the 3 ms duration. As was found with the 500 Hz frequency, the application of high rate and long stimulus duration again finds mean SP amplitude that is significantly larger when compared to slow rates, suggesting the use of high rate to elicit a larger SP when long duration stimuli are utilized. Again, the opposite trend was found with the use of short duration stimuli where we observed mean SP amplitude decreasing with increasing rate.

## Discussion

The use of ECochG in the assessment of the auditory system has garnered a great deal of evaluation in terms of protocol, parameters, and methodology. While ECochG measurements to tone bursts have been studied for its potential usefulness in objectively assessing Ménière's disease (Gibson, 1993, 2009; Ferraro and Krishnan, 1997; Wuyts et al., 1997; Conlon and Gibson, 2000; Iseli and Gibson, 2010), a condition defined by endolymphatic hydrops (Merchant et al., 2005; Nadol, 2010), fewer studies have examined the usefulness of slow repetition rates (Levine et al., 1992; Ferraro et al., 1994a; Margolis et al., 1995). Ours was a study on a novel assessment of ECochG measurements to tone bursts analyzed with the CLAD technique to quantify SP amplitude at various stimulus repetition rates. Normative data was obtained across frequency, stimulus repetition rate, and stimulus duration to understand the effects of these parameters on the SP.

### SP amplitude

Our study is rooted in the assumption that SP originating from hair cells will sustain throughout the duration of the measurement because hair cells do not habituate, while SP originating from neural excitation will decrease in amplitude because of neural habituation. We found that trends in SP amplitudes to various tone burst repetition rate and durations for both 500 and 2,000 Hz. In particular, SP amplitude was significantly larger for the highest stimulus repetition rate, 58.59/s compared to the slower rates, 7.1 and 19.53/s. Additionally, SP amplitude was significantly larger for the shortest tone burst duration, 3 ms, compared to longer durations, 12 and 6 ms. SP amplitudes were significantly larger for 2,000 Hz than 500 Hz for each stimulus repetition rate and duration.

Overall, the longer duration stimuli evoked larger SP amplitudes with increasing stimulus repetition rate. However, this trend was reversed with the use of short duration stimuli (3 ms), in which mean SP amplitude to 3 ms duration decreased as repetition rate increased. SP amplitudes to 3 ms tone bursts were larger for all repetition rates. While our results are statistically significant, they are not always consistent with that found in previous studies. We flesh out the inconsistencies below in this section.

#### Slow repetition rate

The SP to long duration (12 ms), slow rate (7.1/s) tone bursts had negative amplitude, with −0.067 μV to 500 Hz and −0.042 μV to 2,000 Hz. The direct comparison of the current SP amplitude measures to published research is difficult due to distinct differences in recording and stimulus parameters, as well as differences in methods used to quantify the SP amplitude. Wuyts et al. (1997) performed a meta-analysis of ECochG measurements to click and tone burst stimuli and found that too few reports exist to extract normative SP amplitude data from tone bursts stimuli. Nevertheless, Wuyts et al.'s meta-analysis found a trend that SP from near-baseline levels generally have positive, not negative, amplitudes. Our approach to assigning the non-inverting and inverting electrodes resulted in positive going SP amplitudes, which is consistent with Wuyts et al. (1997) report of negative SP amplitudes. Our findings are consistent with those reported by Ferraro et al. (1994a), though precise stimulus parameter differences exist. Ferraro et al.'s results to 90 dBnHL, 11.3/s, and 2-10-2 duration can be generally compared to our SP amplitudes to slow rate, long duration tone bursts. Ferraro et al. (1994a) found mean SP amplitude values of 0.19 μV to 500 Hz and 0.08 μV to 2,000 Hz, which are comparable to our SP amplitudes when polarity differences from electrode montages are accounted for. While our results are similar to the Ferraro et al. (1994a) study, other reports utilizing long duration tone bursts (15 ms) with slow rate (13/s) obtained larger, positive SP amplitude values in subjects without inner ear disease: Margolis et al. (1995) found respective SP amplitudes of 0.65 and 0.96 μV to 100 and 110 dB SPL 2,000 Hz tone bursts from ears with normal hearing, which are markedly larger than amplitudes found in our study, even when compared to the most positive amplitudes to our 7.1 and 19.53/s stimulus repetition rates.

Our measurements to 500 Hz tone bursts can be informative about the extent to which neural excitation can contribute to recordings made from the auditory periphery. We found oscillations in response waveforms to slower rate, long duration conditions (Figure 2), consistent with the contribution of neural excitation that is phase locked to this low frequency stimulus (Lichtenhan et al., 2013, 2014; Chertoff et al., 2015). Oscillations decreased with increasing stimulus repetition rate, further supporting the interpretation of the neural origin of this waveform component, as auditory nerve fibers cannot respond to high-rate stimuli while in their refractory period. As such, it is possible that the oscillations occurring in the slow rate, long duration 500 Hz recordings are contributing to the mean SP amplitude results obtained in the study.

SP amplitude was measured from one pre-defined midpoint along the waveform for all rates and participants and the measurements did not take into account variations associated with the peaks and troughs of the oscillations. As SP amplitudes collected were small, these oscillations may have had a significant impact on the collected amplitude data. For example, the midpoint occurring at a peak of the oscillation for one recording and at a trough for another had the potential to influence the SP amplitude values obtained.

**Figure 7:**
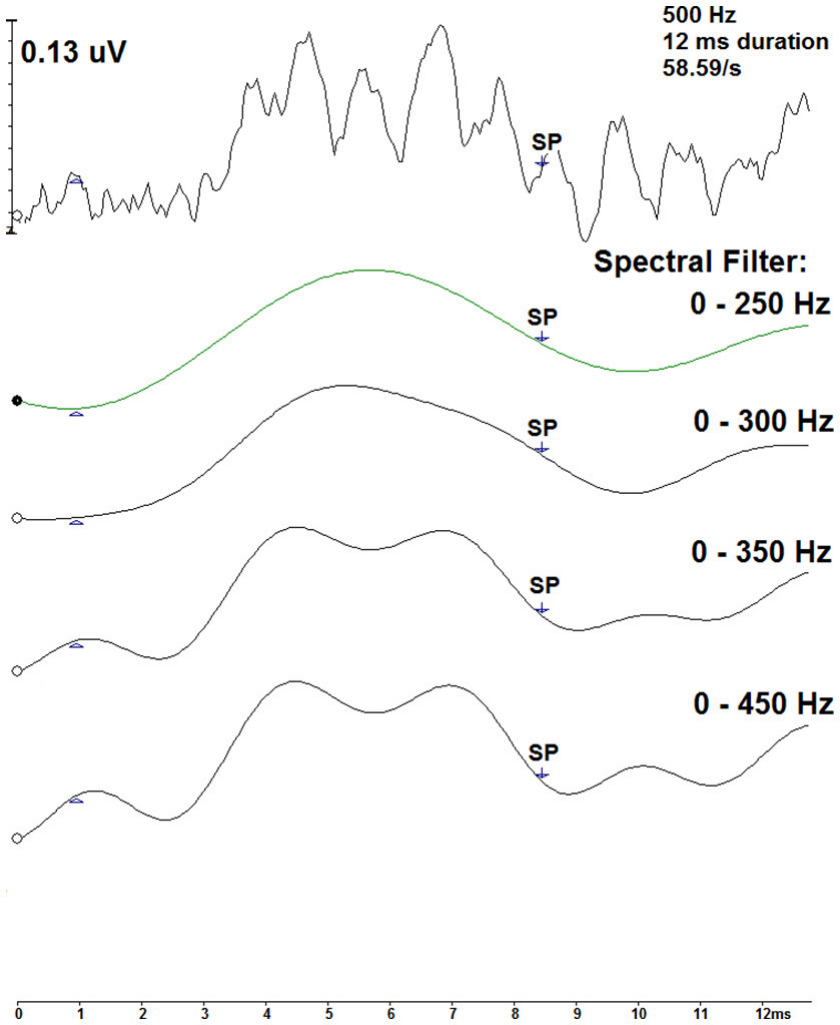
Spectral filtering of a CLAD deconvolved tone burst ECochG waveform (top) for the 500 Hz, 12 ms, 58.59/s condition. Displayed in descending order, four spectral band pass filters were applied: 0–250, 0–300, 0–350, and 0–450 Hz. SP indicates the pre-defined midpoint of the 12 ms duration.

A technique to reduce the contribution of the oscillations within the 500 Hz recordings is to filter the measurements with a low-pass cut-off frequency. Using the Intelligent Hearing Systems software, spectral filtering was applied offline to a single measurement to evaluate this method as a technique to examine the 500 Hz recordings. Figure 7 displays this technique for a 12 ms, 58.59/s deconvolved trace across four spectral filters: 0–250 Hz, 0–300 Hz, 0–350 Hz, and 0–450 Hz. As more filtering was applied, the smoother the resultant waveform. The labeled SP indicates the pre-defined midpoint for the 12 ms stimulus duration. Filtering the measurements is a possible technique to improve SP detection.

#### High repetition rate

Our negative SP amplitudes to slow repetition rate, 12 ms stimulus duration tone bursts contrast the positive amplitudes we measured to higher rates. SP amplitudes to higher rate (58.59/s), long duration (12 ms) tone bursts were significantly larger than those to slower rates (7.1 and 19.53/s) to both tone burst frequencies. Oscillations in responses to high rate, 500 Hz tone bursts were reduced and a positive mean SP amplitude of 0.028 μV was collected for the highest rate. There were no significant differences found among SP amplitudes to 6 ms 500 Hz tone bursts of various repetition rates. These results can inform clinicians and basic investigators on the appropriate parameters needed to assess low-frequency function: use long duration stimuli with a high repetition rate to quantify non-neural SP to 500 Hz.

Also our results showed that SP amplitudes tended to increase with increasing stimulus repetition rate with the use of 2,000 Hz, long duration tone bursts, a common finding in previous studies. Wilson and Bowker (2002), for example, studied ECochG measurements to clicks ranging from 7.1 to 151.1/s, albeit without the CLAD technique. They found that SP amplitudes increased in response to higher stimulus repetition rates. However, their SP amplitudes were overall reduced because of poor frequency specification to their click stimuli and poor morphology and overlying responses that result from not using the CLAD technique. These findings highlight the usefulness of tone burst stimuli and the CLAD technique for measurements to high stimulus rates.

#### Stimulus level and recording location

We made SP amplitude measurements to higher stimulus repetition rates, shorter stimulus duration, and, perhaps more importantly, to low stimulus intensity. DC responses are generally thought to originate from higher level stimuli that probe the asymmetric regions of the sigmodial, saturating, non-linear function that can describe the transfer of sound from mechanical to electrical mediums in the inner ear. We avoided high-intensity stimuli because of our lengthy test sessions, and presented both the 500 and 2,000 Hz tone bursts at 75 dB nHL (107 dB SPL), a level which may resulted in lower SP amplitudes. It is possible that our lower levels may have evoked a larger SP had we used a transtympanic approach, but a transtympanic approach would increase the measured amplitude of all DC origins, albiet hair cell or neural. Indeed, amplitudes from an extratympanic approach can be ~4–10 times smaller than those from transtympanic approach (Ferraro et al., 1994b; Haapaniemi et al., 2000). Direct microscope visualization for uniform electrode placement on the umbo may provide uniformity in measurements, but straightening the ear canal with the electrode in place can be painful and direct microscopic visualization is challenging because the white cotton tipped electrode soaked in gel reflects a light that obstructs the visualization of electrode placement. The most common way to identify electrode placement is after measurements are done and remaining electrode gel and irritation is visualized with otoscopy. Smith et al. (2016) found that electrode placement mostly affects measurements from low-frequencies when an insert-earphone is used, a possible influence on our measurements to 500 Hz.

### High repetition rate and continuous loop averaging deconvolution

Our study demonstrated the use of the CLAD technique applied to ECochG measurements to tone bursts. Several studies have also demonstrated the use of the CLAD technique to ECochG measurements, but those studies focused on the use of click stimuli (Bohorquez et al., 2006, 2009; Bextermueller, 2015; Dixon, 2015; Kaf et al., 2017). Measurements to stimulus repetition rates up to 234.38/s were successfully deconvolved allowing for clear observation of the SP and the AP within the recordings. This novel finding supports the use of CLAD with responses evoked using tone burst stimuli. With close monitoring of the maximum repetition rate in the CLAD sequence, CLAD can be applied to test SP amplitudes at high rates which were previously limited due to the overlain responses.

### Limitations and future studies

Our results cannot be generalized outside the specific recording analysis techniques. Currently, there is no standardized tone burst parameters for ECochG approaches across research institutions. This is a double edged sword making direct comparison from one study to another quite challenging, but does not restrict investigators' creative use of stimulus parameters to study and understand normal and diseased ears.

We subjectively measured SP amplitudes at mid-point that was relative to a fixed waveform onset to mediate uniform SP amplitude measurement across participants. A limitation of this approach was that SP onset did indeed vary between participants. Our SP amplitude measures may thus have variations that were untethered to a gold standard for the onset of SP measurements. Future research could reassess our data to determine how various definitions of SP onset influences results.

The most pressing research to address in future work on this topic is to use legitimate DC-coupled recordings of SP measurements and validate our interpretations in animal models where the neural origins of SP amplitude measures can be manipulated. Injection of neurotoxic solutions into the cochlear apex is a new approach that can treat the entire length of the cochlear spiral (Lichtenhan et al., 2016). Using the stimulus and analysis approaches of our current study in animals where the apical injection technique can be applied could quantify the extent to which neural excitation contributes to our interpretation of data presented here.

## Conclusion

We collected normative SP amplitude from females with normal hearing using extratympanic ECochG, tone burst stimuli, and a CLAD analysis technique. SP amplitude measures to 2,000 Hz, long duration stimuli increased with increasing repetition rate, as did SP amplitudes to 500 Hz with the longest stimulus duration (12 ms) and highest stimulus repetition rate (58.59/s). These increased amplitude measures are consistent with SP origins from hair cell responses, not neural excitation, and suggest that high stimulus repetition rate could be used to minimize neural contributions to SP measures. SP amplitude measures to our shortest stimulus duration (3 ms) were consistent with marked contribution of neural excitation, thus identifying a stimulus condition to use when an SP measurement originating from neural responses is desired. Our study also demonstrated the use of the CLAD technique with ECochG measurements to tone bursts presented with high stimulus repetition rates. While the use of tone burst stimuli limited our stimulus repetition rate to 234.38/s, the deconvolved waveforms nevertheless show that the CLAD technique can be used with frequency-specific stimuli. Overall, this research was a step toward understanding how varying stimulus parameters can be used to advance our understanding of the origins of SP amplitude measures, an important step for advancing the use of ECochG in diagnosis of Ménière's disease that mainly affects low frequency hearing early in the disease process.

## Author contributions

AK, WK, RD, JL, JF: Meet all criteria for authorship.

### Conflict of interest statement

The authors declare that the research was conducted in the absence of any commercial or financial relationships that could be construed as a potential conflict of interest.
